# Evaluation of the Impact of the Cancer Therapy Everolimus on the Central Nervous System in Mice

**DOI:** 10.1371/journal.pone.0113533

**Published:** 2014-12-01

**Authors:** Martine Dubois, Vadim Le Joncour, Marie-Christine Tonon, Youssef Anouar, François Proust, Fabrice Morin, Pierrick Gandolfo, Florence Joly, Pascal Hilber, Hélène Castel

**Affiliations:** 1 Inserm U982, Laboratory of Neuronal and Neuroendocrine Communication and Differentiation (DC2N), Astrocyte and Vascular Niche, University of Rouen, Mont-Saint-Aignan, France; 2 PRES Normandie Université, Institute of Research and Biomedical Innovation (IRIB), University of Rouen, Mont-Saint-Aignan, France; 3 North-West Cancéropole (CNO), Lille, France; 4 EA4700, Laboratory of Psychology and Neurosciences of Cognition and Affectivity (PSY-NCA), University of Rouen, Mont-Saint-Aignan, France; 5 Centre François Baclesse, Caen, France; 6 Centre Hospitalo-Universitaire, Caen, France; IIT Research Institute, United States of America

## Abstract

Cancer and treatments may induce cognitive impairments in cancer patients, and the causal link between chemotherapy and cognitive dysfunctions was recently validated in animal models. New cancer targeted therapies have become widely used, and their impact on brain functions and quality of life needs to be explored. We evaluated the impact of everolimus, an anticancer agent targeting the mTOR pathway, on cognitive functions, cerebral metabolism, and hippocampal cell proliferation/vascular density in mice. Adult mice received everolimus daily for 2 weeks, and behavioral tests were performed from 1 week after the last treatment. Everolimus-treated mice displayed a marked reduction in weight gain from the last day of the treatment period. *Ex vivo* analysis showed altered cytochrome oxidase activity in selective cerebral regions involved in energy balance, food intake, reward, learning and memory modulation, sleep/wake cycle regulation, and arousal. Like chemotherapy, everolimus did not alter emotional reactivity, learning and memory performances, but in contrast to chemotherapy, did not affect behavioral flexibility or reactivity to novelty. *In vivo* hippocampal neural cell proliferation and vascular density were also unchanged after everolimus treatments. In conclusion, two weeks daily everolimus treatment at the clinical dose did not evoke alteration of cognitive performances evaluated in hippocampal- and prefrontal cortex-dependent tasks that would persist at one to four weeks after the end of the treatment completion. However, acute everolimus treatment caused selective CO modifications without altering the mTOR effector P70S6 kinase in cerebral regions involved in feeding behavior and/or the sleep/wake cycle, at least in part under control of the solitary nucleus and the parasubthalamic region of the hypothalamus. Thus, this area may represent a key target for everolimus-mediating peripheral modifications, which has been previously associated with symptoms such as weight loss and fatigue.

## Introduction

Although the emergence of potent anticancer agents has improved patient survival, there is increasing evidence that both cancer and its treatments can induce cognitive dysfunctions that affect daily quality of life. Patients receiving chemotherapy report attention and concentration alterations, visual and verbal memory deficits, and slowing of psychomotor processing (referred to as “chemofog” or “chemobrain”) which can persist for several years after treatment completion [1]. In recent years, targeted agents have been increasingly used in cancer treatment, and previous reports suggest that some of them may permeate the blood-brain barrier and act directly in the brain, affecting cerebral angiogenesis and functioning [2]. Consistent with this hypothesis, administration of bevacizumab in patients with metastatic colorectal cancer and sunitinib in patients with metastatic renal cancer resulted in several reported cases of posterior leukoencephalopathy [3], [4]. Furthermore, an unexplained fatigue or asthenia, associated with targeted cancer treatments, that cannot be counteracted by rest or sleep may severely affect both cognitive function and quality of life [5].

The phosphatidylinositol-3-kinase (PI3K)/AKT/mammalian target of rapamycin (mTOR) signaling cascade is a key molecular target for cancer treatment [6]. mTOR signaling components are expressed at high levels in several areas of the brain [7], [8], and the mTOR pathway is known to be involved in various neurobiological processes, including neurite outgrowth [9], axon regeneration [10], myelination [11], and cellular metabolism [12]. In particular, mTOR has been shown to be a central regulator of cell growth and is controlled by a large number of signals including nutrients such as glucose and amino acids, and growth factors such as insulin and IGF-1. mTOR activation also stimulates protein synthesis and cellular hypertrophy in various cells and organs. In addition, mTOR activation is involved in hippocampal synaptic plasticity and learning and memory processes *via* protein synthesis [13]. For example, inhibition of mTOR activity by rapamycin has been shown to block inhibitory avoidance long-term memory [14] and to impair auditory [15], fear [16], [17], and spatial memory consolidation [18]. Moreover, genetic defects in Ras/Erk/PI3K/mTOR signaling pathways may be causally linked to several human genetic disorders classified as neuro-cardio-facial-cutaneous and hamartoma syndromes, and thus may be responsible for cognitive impairments [19]. Thus, it may be proposed that long-term administration of mTOR inhibitors occurring in cancer treatment could affect brain functions involved in cognition and/or metabolism.

Everolimus (Afinitor, Novartis, Basel, Switzerland), an orally administered rapamycin derivative, directly blocks the kinase activity of the raptor/mTOR complex (mTORC1) *via* binding to the FKBP-12 and thus forming an inhibitory complex with mTOR [20]. This type of mTOR inhibitor is well characterized for anti-neoplastic properties, *i.e.* inducing decrease of tumor cell growth, proliferation, and angiogenesis *in vitro* and *in vivo* [21]–[23]. However, general side effects including fatigue, edema, asthenia, pyrexia, mucosal inflammation, anorexia, decreased weight and pain have been reported [24]–[27]. More specifically, central nervous system (CNS) side effects including headache/migraine or dysgeusia, have been also described [26].

The goal of the study was to evaluate the potential cognitive functioning–impairment likely to be induced by targeted therapies such as everolimus by using a validated mouse behavioral model [28]. We particularly gained attention in the dose used in order to be as much as possible in the cancer patient treatment conditions. Indeed, based on pharmacokinetic/dynamic studies [29]–[31] the most adapted and indicated dose in the advanced hormone receptor-positive, HER2-negative breast cancer, advanced neuroendocrine tumors of pancreatic origin, advanced renal cell carcinoma, renal angiomyolipoma with tuberous sclerosis complex (TSC) is 10 mg/daily, but in case of toxicity, the suggested dose is 5 mg/daily. Also, a number of studies were conducted in animal models with this 5 mg/daily administration [22], [32], [33] and studies demonstrated that this everolimus dose is orally active in mice and that steady state achieved within two weeks with daily dosing [22]. This 5 mg/kg/d schedule appears to be the minimal concentration for the maximal efficient dose with no apparent toxicity.

The potential long-lasting effects of everolimus treatment on mice cognition and neurobiological processes were evaluated using hippocampal- and frontal cortex-dependent behavioral tasks and on hippocampal cell proliferation, or vascular niche density respectively. Its acute impact on neural cell activity was investigated *ex vivo via* assessment of regional brain cytochrome oxidase activity.

## Material and Methods

### Animals and ethic statements

Male C57BL/6J Rj mice (Janvier, Le Genest Saint Isle, France) 7 weeks of age were housed under controlled standard environmental conditions: 22±1°C; 5 animals per cage; 12 hours/12 hours light/dark cycle (light on: 00:00); water and food available *ad libitum*. During the 2-weeks adaptation period, animals were handled daily for weight monitoring. Treatment administration began when mice were 9 weeks of age. Mice were weighed daily from the day of the 1st treatment ingestion to the 36th day after the last administration. The weight of animals before the 1st injection (D0) was used as the basis value to calculate the weight gain throughout the experiment. All procedures were performed in accordance with the French Ethical Committee as well as the guidelines of European Parliament directive 2010/63/EU and the Council for the Protection of Animals Used for Scientific Purposes. This project was approved by the “Comité d'Ethique NOrmandie en Matière d'EXpérimentation Animale” CENOMEXA under the National Committee on Animal Experimentation, and received the following number N/12-11-12/35/11-17. Animal manipulations were carried out under the supervision of an authorized investigator (H. Castel; authorization no. 76.98 from the Ministère de l'Alimentation, de l'Agriculture et de la Pêche).

### Drug administration

A microemulsion of everolimus formulated at 2% (w/v), provided by Novartis (Rueil-Malmaison, France), was dissolved in 0.9% NaCl and administered daily by oral gavage (5 mL/kg) *via* a feeding needle (Fine Science Tools, Heidelberg, Germany) at 5 mg/kg for 14 consecutive days [34].

### Cytochrome oxidase activity and brain metabolism

Mice that received vehicle (*n* = 8) or everolimus (*n* = 9) during 2 weeks were immediately euthanized after the last day of treatment to study cytochrome oxidase activity in various cerebral regions. Mice were anesthetized using isofluorane, decapitated, and their brains rapidly removed 24 hours after the last day of treatment, frozen in 2-methylbutane (Sigma-Aldrich, San-Quentin Fallavier, France) at −30°C and stored at −80°C until use. Cryostat sections, each 30 µm thick, were cut from Bregma +3.20 mm to −6.48 mm and stored at −20°C until processing. For each animal, 8 batches of 41 consecutive slices were taken, such as on one slide 2 sections were separated by 240 µm.

All brain slices of one batch per animal were processed simultaneously under the same conditions. The protocol was adapted from previous publications [35], [36]. Sections were incubated in the dark (37°C, 50 minutes) in 0.1 M phosphate-buffered saline (PBS) containing 120 mg horse-heart cytochrome c, 24 g sucrose, 300 mg DAB-4-HCl, and 108 mg catalase (Sigma-Aldrich, Saint-Quentin Fallavier, France) per 540 ml. Slices were washed (5 min) with cold buffer (10% sucrose in PBS), immersed for 30 minutes in a 10% formalin/buffer solution, and washed twice (5 minutes) in buffer before dehydration. Sections were then cover-slipped with Eukitt (VWR International, Strasbourg, France) and microscopically examined.

The product of the histochemical reaction (oxidized DAB) was visible with optical microscopy. In each area of interest, its staining intensity was measured by densitometric analysis by means of a computer-assisted image analysis workstation (SAMBA Technologies, Meylan, France) [37]. For each area studied, a minimum of 2 slices per animal were used, depending of the anteroposterior length of the region, with measurements performed bilaterally when possible. The background was subtracted for each slide. The different cerebral regions were identified by means of the Franklin and Paxinos [38] mouse brain atlas.

### Behavioral testing

In total, 14 mice received everolimus treatment and 12 mice received solvent only (vehicle group) during 2 weeks. Behavioral testing began 7 days after the last treatment administration and lasted 23 days (Fig. S3A). All experiments were conducted between 13:00 and 18:00, during the beginning of the animals' active phase.

Cognitive functions were evaluated using the Morris water maze, in which mice are required to escape from a water basin by finding a platform placed in the pool [39]. A cylindrical tank (diameter 93 cm, height 45 cm) was filled with water (maintained at 23±1°C) to a height of 40 cm, made opaque with white, inert, aqueous acrylic emulsion (Accusol OP 301, Viewpoint, France). The tank was placed in an illuminated room (50 lux at the central surface of the pool) with extra-maze cues on the walls. The water maze was divided into four virtual quadrants: north-west (NW), north-east, south-east, and south-west, and animal behaviors were video tracked. On day 1, animals were familiarized with the pool and their motivation and visuo-motor abilities evaluated. An escape platform (diameter 9.7 cm) was placed in the tank center, emerging 1 cm above the water surface and with a small black ball fixed on it in order to facilitate its viewing on the part of the animals. Mice were placed on the platform for 20 seconds. Immediately afterwards, 1 session comprising four 60-second trials (inter-trial interval: 30 minutes) was conducted. Animals were placed, facing the wall, at 1 of 4 start locations (north, south, east or west) and allowed to swim to the visible platform for a maximum of 60 seconds. Mice not finding the platform were placed manually upon it for 20 seconds. On days 2–5, spatial learning abilities were evaluated in a training phase of 4 daily trials, maximum 60 seconds each, in which the escape platform was placed in the NW quadrant, immersed 1 cm below the water surface. 2 hours after the final trial of the last day of the training period, a probe test was conducted by removing the platform and allowing animals to swim for 60 seconds, with time spent in the previously correct quadrant (NW) measured. Spatial memory abilities were evaluated in a retrieval phase on day 10, using a single session of 4 trials under the same conditions as the training phase. Learning plasticity was evaluated in a transfer phase over 4 successive days (4 trials/day, maximum 60 seconds each) by daily changing the hidden platform location. Distance crossed and escape latency were measured.

### Hippocampal cell proliferation, vascular density and phosphorylated P70S6 kinase immunolabeling

5–bromo-2-deoxyuridine (BrdU)-labeled cells inside and outside the subgranular zone of the hippocampus (SGZ) were counted. To label adult-generated cells in the dentate gyrus of the hippocampus, 6 intraperitoneal injections (50 mg/kg) of BrdU (Sigma-Aldrich, Saint-Quentin Fallavier, France) were administered during the last days of the treatment period in mice not undergoing behavioral evaluation (Fig. S3C). The day following the last gavage, and 3 hours post-BrdU injection, mice were anesthetized using isofluorane, decapitated, and their brains rapidly removed, frozen in 2-methylbutane (Sigma-Aldrich, Saint-Quentin Fallavier, France) at −30°C and stored at −80°C until use. Brains were cut with a cryostat into serial coronal sections (thickness 20 µm) from the anterior part of the dorsal hippocampus (anteroposterior, 1.20 mm from the Bregma −1.44 mm). Every 12th section, each separated by 240 µm, was mounted on slides coated with gelatin-chrome alum and stored at −20°C until processing.

Six hippocampal sections from each of 4 animals per group were stained simultaneously for BrdU visualization. Brain slices were fixed (4% paraformaldehyde in PBS, washed 3×5 minutes with PBS pH 7.4, and incubated (2N HCl; 45°C; 45 minutes) to denature DNA. Sections were then washed (PBS, 3×5 minutes), incubated for 1 hour in blocking solution containing 1∶50 normal donkey serum, 1% bovine serum albumin, and 0.3% Triton X-100 (VWR International, Strasbourg, France) in PBS, and incubated overnight at 4°C with sheep anti-BrdU immunoglobulin G (IgG; Abcam, Paris, France) at 1∶400 in blocking solution. Subsequently, sections were washed (PBS, 4×5 minutes) and incubated (2 hours, room temperature) with Alexa 488-conjugated donkey anti-sheep IgG (Invitrogen, Boulogne-Billancourt, France) at 1∶400 in PBS. Rinsed sections were cover slipped with mowiol. Sections were examined on a Nikon Eclipse E600 microscope (Nikon Instrument, Champigny-sur-Marne, France) interfaced with the Mercator software (ExploraNova, La Rochelle, France). A modified unbiased stereological method was used to count BrdU-positive cells in the SGZ of the dentate gyrus and in the area outside the SGZ [40], [41]. The number of stained cells in 6 sections was counted bilaterally, and, as every 12th section was used, this number was multiplied by 12. All counts were done by an investigator blinded to the treatment group.

For investigation of hippocampal vascular niche density, brain sections were fixed, washed, and incubated as described above. The primary antibody was rabbit anti-IQGAP1(H-109) (Santa Cruz Biotechnology, Inc., Tebu-bio, Le-Perray-en-Yvelines, France) diluted 1∶400 in blocking solution, and the secondary antibody was Alexa 488-conjugated donkey anti-rabbit IgG at 1∶400 in PBS. Sections were counterstained with nuclear marker DAPI (1 µg/mL). Immunofluorescence was observed under a confocal microscope and IQGAP1-labeled structures in the SGZ assessed in 3 sections separated by 240 µm (between Bregma −1.68 mm and −2.16 mm).

To investigate the active forms (phosphorylated) of P70S6 kinase (p-P70S6K), brain sections of vehicle and everolimus-treated mice were fixed, washed, and the p-P70S6K expression was measured by means of the primary antibody anti-p-P70S6K (Abcam ab60948, Paris, France) diluted 1∶200 in blocking solution, and the secondary antibody was Alexa 488-conjugated donkey anti-rabbit IgG at 1∶400 in blocking solution. P-P70S6K-labeled structures were assessed in 2 or 3 sections separated by 240 µm (between Bregma −0.98 mm and −1.70 mm for arc, Re, Rh, submed, VM; between −1.22 mm and −1.94 mm for EP, Ect and PRh; between −2.06 mm and −2.54 mm for PSTh) with Nikon Eclipse E600 microscope (Nikon Instrument, Champigny-sur-Marne, France) interfaced with the imaging software NIS-Elements.

### Culture of neural stem cells and endothelial cells

For neural stem cell (NSC) culture, brain from C57BL/6J Rj newborn mice were washed in trypsin. The cell suspension was centrifuged at 100 G for 4 minutes, and cells were placed into fresh serum-free medium. This medium consisted of Neurobasal (100 mL, Gibco, Saint-Aubin, France) supplemented with 5 mM HEPES buffer (Sigma-Aldrich, Saint Quentin Fallavier, France), 1 mL B27 and 1 mL N2 growth supplement (Gibco), 20 µL epidermal growth factor (20 ng/mL; Sigma-Aldrich, Saint Quentin Fallavier, France), 10 µL basic fibroblast growth factor (10 ng/mL; Sigma-Aldrich, Saint Quentin Fallavier, France), and 7.32 µL heparin (Sigma-Aldrich, Saint Quentin Fallavier, France) and was added with 33 mM glucose. Cells were plated at a density of 100 viable cells per microliter in this medium and were cultured in 25 cm^2^ flasks at 37°C in a 5% CO_2_–95% air humidified incubator. On day 7, well-developed neurospheres were collected and digested in trypsin for 10 minutes at 37°C. Secondary neurospheres (P_1_) and later passages were generated by mechanical and enzymatic dissociation of primary neurospheres. Cells were then incubated in the absence or presence of vehicle or everolimus for 24 to 48 hours. Using a 4× objective of a Nikon (Nikon Eclipse TS100, Kingston, England) inverted microscope, we measured the mean diameter of 20 randomly chosen neurospheres per well (>40 µm in diameter) in the visual field in 4 different areas of 3 wells of 24-well culture plates.

For endothelial cell culture, we used polyoma virus middle-sized tumor antigen (mT)-transformed mouse brain capillary endothelial cell line (bEND.3, obtained from Dr D. Gorecki, Portsmouth University, England). Cells were cultured in high glucose (4.5 g/L)-containing Dulbecco's modified Eagle's medium supplemented with 1.5 g/L sodium bicarbonate, 100 U/mL penicillin, 100 µg/mL streptomycin, and 10% fetal bovine serum (FBS) at 37°C with 5% CO_2_. To determine cell survival and proliferation, cells were plated in triplicate in multiple sets of 12-well culture plates. Incubation ± vehicle/everolimus for 24–48 hours was in FBS-free medium. Cell proliferation was quantified by electronically counting cell numbers (Z2, Beckman Coulter, Villepinte, France).

### Statistical analysis

Body weight gain and spatial learning performances were analyzed by 3-ways ANOVA with repeated measurements followed by least significant difference (LSD) post hoc analyses when they were necessary. For probe test of the Morris water maze, time spent in the quadrant where the platform was located was compared with that predicted by chance by means of χ^2^ tests. Other behavioral data, immunohistochemical and cytochrome oxidase activity data were analyzed with Student *t* tests. These data were analyzed with Statistica© 5.1. Data of the hippocampal cell proliferation and data of the culture studies were analyzed with the nonparametric Mann-Whitney U-test and ANOVA of Kruskal-Wallis followed by Dunn's tests with GraphPad Prism 5. For all statistical tests, the threshold of significance was set at *p* ≤.05.

## Results

### Everolimus-induced weight gain alterations

**Figure 1:**
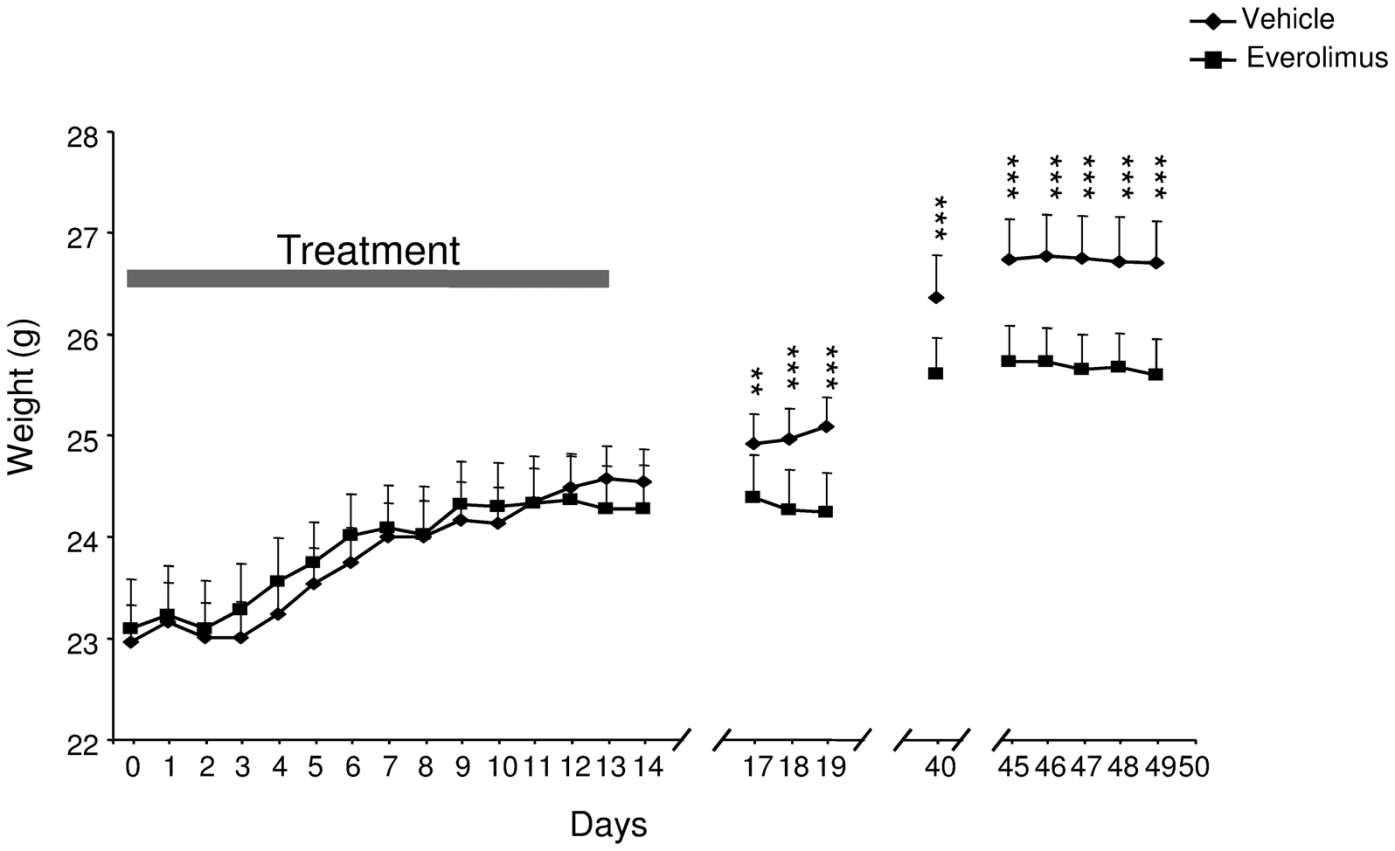
Impact of everolimus treatment on the mean body weight of mice. Body weight of vehicle- and everolimus-treated mice was evaluated at long term after the end of the treatment period (gray bar). Mice received vehicle or everolimus once a day during 14 continuous days. Bars represent standard error of the mean. ANOVA, Treatment x Day interaction *p*<.001 followed by LSD post hoc: **p<.01, ***p<.001.

We first evaluated the consequences of a chronic mTOR inhibition on body weight in mice. Administration of everolimus (5 mg/kg) daily to young mice by gavage during 2 weeks produced no apparent morbidity and/or mortality. Whereas the control (emulsion) group continued to gain weight over the course of the experiment, the everolimus-treated mice had reduced weight (treatment × day interaction: F_23,552_ = 8.29, *p*<.001) from the 17rd day after the introduction of the mTOR inhibitor, and continued to weigh less throughout the following experimental period (LSD post hoc *p*<.01; Fig. 1).

### Short-term impact of everolimus on cerebral cytochrome oxidase activity and P70S6 kinase phosphotylated form

**Figure 2:**
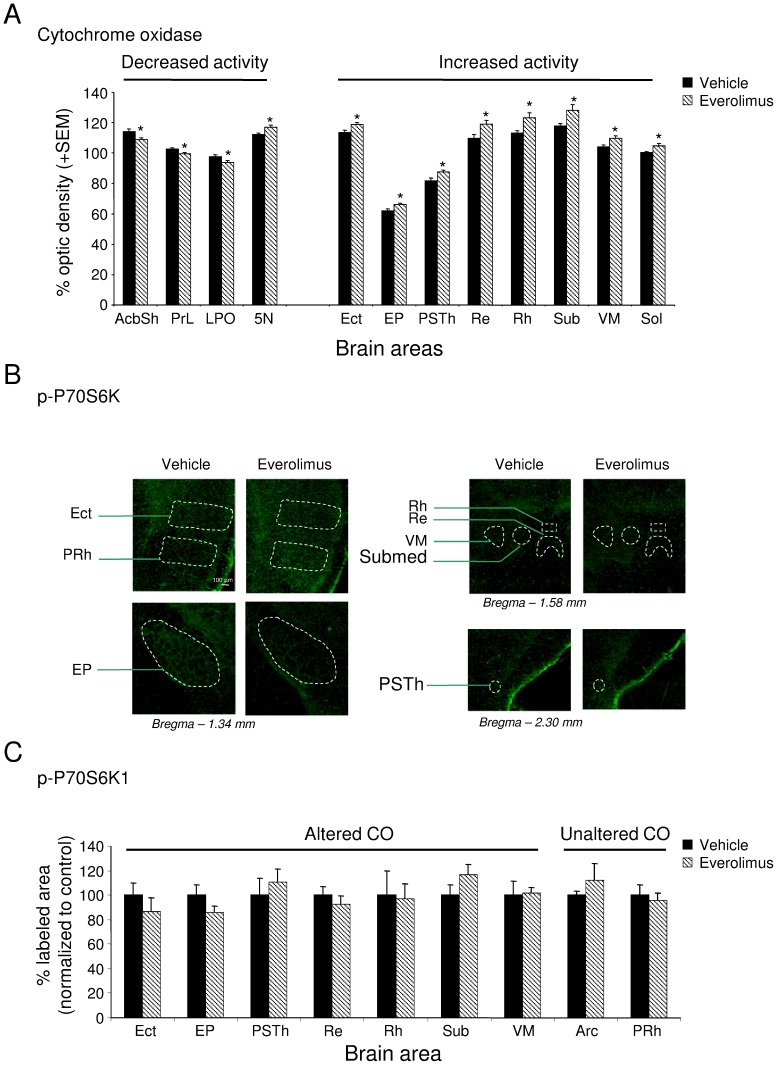
Phospho-P70S6K labeling in brain regions of everolimus and vehicle mice. (a) CO activity in selective vehicle- and everolimus-treated mice brain areas. (b) Representative images of Phospho-P70S6K (p-P70S6K) immunolabeling in cortex, entopeduncular nucleus, thalamus and hypothalamus of vehicle or everolimus-treated mice. (c) Mean percent of P-P70S6K-labeled surface (normalized to vehicle) (+SEM) in the different brain areas. Arc: arcuate nucleus; Ect: ectorhinal cortex; EP: entopeduncular nucleus; PRh: perirhinal cortex; PSTh: parasubthalamic nucleus; Re: reuniens thalamic nucleus; Rh: rhomboid thalamic nucleus; Submed: submedial thalamic nucleus; VM: ventromedial thalamic nuleus.

In order to evaluate the impact of the mTOR inhibitor on cerebral cell metabolism, a histochemical analysis revealing cytochrome oxidase activity has been performed in brain slices of control and treated mice (Table 1). In everolimus-treated mice, 4 days after the end of the treatment period, a significant decrease (*p*<.05) of cytochrome oxidase activity was present in the shell part of the accumbens nucleus (t_13_ = 2.52), the prelimbic cortex (t_15_ = 2.20), the lateral preoptic area (t_14_ = 2.33) and the motor trigeminal nucleus (t_13_ = 2.38). Moreover, everolimus treatment resulted in increased metabolic activity in the ectorhinal cortex (t_14_ = −2.61), the entopeduncular nucleus (t_13_ = −2.27), the parasubthalamic nucleus of the hypothalamus (t_14_ = −2.91), the solitary nucleus (t_13_ = −2.49), and in the thalamic reuniens (t_14_ = −2.48), rhomboid (t_14_ = −2.31), submedius (t_14_ = −2.30), and ventromedial (t_14_ = −2.56) nuclei (all *p*<.05) (Fig. 2A and Table 1). In order to investigate the potential direct or indirect inhibition of mTOR in these brain areas, the activity of a downstream effector of the mTOR pathway was assessed by immunostained levels of the phospho-P70S6 kinase (p-P70S6K) in various brain areas exhibiting or not CO metabolic changes. The treatment did not significantly modify P70S6K-positive labeling in the ectorhinal cortex, entopeduncular nucleus, reuniens, rhomboid, ventromedial and submedial thalamic nuclei and the parasubthalamic nucleus exhibiting modified CO activities, as well as in perirhinal cortex and arcuate hypothalamic nucleus showing no changes in CO activities (Fig. 2B and C).

**Table 1:** Cytochrome oxidase activity in cerebral areas of vehicle and everolimus-treated mice.

Regions	Vehicle	Everolimus
**Telencephalon**		
** Amygdala**		
**Basolateral**	112.54 (1.70)	115.97 (1.16)
**Basomedial**	101.60 (1.62)	104.77 (1.30)
**Central**	116.16 (1.35)	115.34 (0.85)
**Cortical**	108.68 (2.89)	113.31 (1.64)
**Lateral**	100.04 (1.71)	100.21 (0.47)
**Medial**	102.65 (2.03)	105.05 (1.35)
** Basal ganglia**		
**Accumbens n.**		
**-Core**	110.96 (1.41)	108.66 (1.16)
**-Shell**	114.10 (1.87)	108.97 (0.99)*
**Caudate Putamen**	105.42 (2.74)	105.47 (1.20)
**Globus pallidus**	87.26 (1.43)	88.85 (0.84)
**Ventral pallidum**	98.19 (2.91)	99.58 (1.14)
** Bed n. stria terminalis**	103.36 (1.21)	105.72 (1.31)
** Claustrum**	94.91 (0.83)	95.15 (0.97)
**Cortex**		
** Auditory**	125.65 (2.56)	130.08 (2.59)
** Cingulate**	112.31 (2.71)	109.97 (0.86)
** Dorsal peduncular**	96.46 (1.64)	99.98 (1.30)
** Ectorhinal**	113.38 (1.57)	118.67 (1.31)*
** Entorhinal**	113.36 (1.84)	118.75 (2.60)
** Frontal association**	106.32 (1.64)	106.79 (1.09)
** Infralimbic**	97.94 (0.58)	97.30 (0.86)
** Perirhinal**	111.61 (1.65)	114.40 (0.71)
** Piriform**	98.87 (0.87)	101.85 (1.21)
** Prelimbic**	102.51 (1.10)	99.50 (0.85)*
** Primary motor**	106.51 (1.37)	104.62 (1.36)
** Retrosplenial**	123.56 (1.16)	121.18 (0.85)
** Somatosensory**	118.71 (1.74)	117.23 (1.23)
** Visual**	128.71 (1.97)	132.99 (2.60)
** Dorsal endopiriform**	102.57 (1.29)	104.30 (1.12)
** Diag. band**		
**n. of the horiz. limb**	100.17 (1.76)	97.68 (0.56)
**n. of the vert. limb**	100.91 (3.08)	105.71 (1.04)
** Hippocampal formation**		
**CA1**	115.25 (1.52)	116.18 (0.93)
**CA3**	111.05 (0.41)	113.30 (1.25)
**Dentate gyrus**	109.60 (0.98)	109.71 (1.20)
**Subiculum**	135.15 (1.55)	139.47 (2.62)
** Olfactory tubercle**	108.20 (5.66)	115.22 (3.69)
** Preoptic**		
**Lateral a.**	97.60 (1.09)	93.96 (1.08)*
**Magnocellular n.**	102.27 (2.60)	101.96 (1.38)
**Medial n.**	99.42 (1.15)	99.32 (1.40)
** Septal n.**		
**Lateral**	106.21 (3.54)	106.46 (1.39)
**Medial**	101.56 (3.81)	103.58 (1.98)
**Diencephalon**		
** Entopeduncular**	62.16 (1.28)	65.95 (1.10)*
** Epithalamus**		
**Lateral habenular n.**	124.94 (2.30)	128.72 (2.18)
**Medial habenular n.**	101.25 (1.99)	103.06 (2.98)
** Hypothalamus**		
**Anterior a., anterior part**	108.69 (3.81)	102.84 (0.92)
**Anterior a., central part**	105.71 (3.24)	106.72 (1.77)
**Anterior a., posterior part**	102.26 (1.57)	103.46 (2.38)
**Arcuate n.**	89.40 (3.43)	88.97 (3.69)
**Dorsomedial n.**	101.80 (1.09)	103.16 (1.65)
**Lateroanterior n.**	106.02 (2.81)	100.70 (2.35)
**Lateral a.**		90.73 (1.66)
**Parasubthalamic n.**	81.72 (1.74)	87.67 (1.21)*
**Paraventricular n.**	98.68 (1.40)	99.56 (1.51)
**Peduncular part**	91.79 (2.15)	91.22 (1.93)
**Perifornical n.**	93.73 (2.28)	92.70 (1.97)
**Posterior a., dorsal**	105.77 (3.79)	102.67 (4.55)
**Posterior n.**	104.86 (1.50)	106.51 (2.03)
**Suprachiasmatic n.**	91.51 (4.41)	96.79 (3.92)
**Ventromedial n.**	105.25 (2.66)	104.41 (1.81)
** Medial mammillary n.**	138.23 (2.66)	144.49 (3.90)
** Subthalamic n.**	132.66 (1.88)	131.93 (1.49)
** Thalamus**		
**Anterior n.**		
**-Anterodorsal**	144.31 (2.43)	145.93 (1.93)
**-Anteromedial**	114.68 (1.74)	114.96 (2.47)
**-Anteroventral**	127.96 (1.57)	132.74 (2.09)
**Dorsal lateral genic. n.**	106.06 (1.97)	107.05 (1.49)
**Intralaminar n.**		
**-Centrolateral**	112.21 (1.90)	116.31 (1.88)
**-Centromedial**	118.27 (3.81)	118.46 (3.02)
**-Paracentral**	108.03 (3.35)	107.26 (2.00)
**-Parafascicular**	104.10 (3.31)	109.70 (1.67)
**Laterodorsal n.**	129.54 (2.81)	132.30 (1.96)
**Midline n.**		
**-Paraventricular**	116.20 (2.26)	117.55 (2.74)
**-Paraventricular, ant.**	123.24 (4.31)	125.60 (1.49)
**-Reuniens**	109.64 (2.70)	119.00 (2.59)*
**-Rhomboid**	113.11 (1.62)	123.04 (3.56)*
**Mediodorsal**	129.12 (1.81)	130.42 (1.96)
**Posterior**	108.10 (1.94)	109.29 (1.53)
**Reticular**	93.41 (1.63)	98.07 (1.54)
**Submedius**	117.77 (1.56)	128.02 (3.72)*
**Ventral n.**		
**-Ventrolateral**	115.16 (1.43)	118.83 (1.79)
**-Ventromedial**	104.00 (1.24)	109.65 (1.68)*
**-Ventral posterolateral**	97.83 (1.93)	100.97 (1.09)
**-Ventral posteromedial**	110.85 (2.32)	112.80 (2.19)
** Zona incerta**	106.57 (2.40)	110.03 (0.86)
**Mesencephalon**		
** Inferior colliculus**	126.02 (2.01)	123.32 (2.87)
** Interpeduncular n.**	159.87 (2.23)	168.43 (5.59)
** Mesenc. ret. form.**	98.86 (1.26)	95.53 (1.46)
** Periaqueducal gray**	118.98 (1.21)	119.50 (1.37)
** Red n.**		
**Magnocellular part**	112.03 (2.06)	115.13 (3.07)
**Parvocellulart part**	104.66 (2.62)	110.54 (2.97)
** Substantia nigra**	110.48 (2.24)	113.25 (2.71)
** Superior colliculus**	116.87 (2.13)	120.36 (2.83)
**Metencephalon**		
** Lateral lemniscus**		
**Intermediate n.**	137.60 (3.61)	134.12 (3.51)
**Ventral n.**	144.65 (1.27)	143.11 (3.09)
** Lateral superior olive**	120.39 (6.03)	122.06 (4.00)
** Locus coeruleus**	105.65 (1.23)	106.70 (1.69)
** Parabrachial n.**		
**Lateral**	99.34 (1.67)	96.21 (1.81)
**Medial**	103.23 (2.31)	100.26 (3.33)
** Pontine n.**	115.02 (2.93)	118.41 (1.82)
** Pontine ret. n.**		
**Caudal part**	86.33 (1.15)	84.45 (1.45)
**Oral part**	106.13 (1.28)	105.19 (0.89)
** Raphe n.**		
**Dorsal**	122.78 (2.19)	125.04 (2.13)
**Medial**	113.43 (0.96)	112.64 (2.03)
** Tegmental n.**		
**Dorsal**	137.89 (2.37)	138.54 (3.08)
**Laterodorsal**	116.94 (3.13)	113.76 (1.28)
**Pedunculotegmental**	90.56 (0.89)	91.18 (1.20)
**Reticulotegmental**	100.18 (1.99)	101.81 (2.73)
**Subpeduncular**	97.91 (1.22)	95.06 (1.18)
** Trapezoid body, n.**	91.97 (3.11)	93.58 (2.02)
** Trigeminal n.**		
**Motor**	112.20 (0.91)	107.67 (1.58)*
**Principal sensory**	117.21 (1.70)	117.04 (1.59)
**Myelencephalon**		
** Solitary n.**	100.39 (0.51)	104.83 (1.39)*
** Cochlear n.**		
**Dorsal**	147.11 (2.16)	147.40 (2.69)
**Ventral**	118.03 (5.03)	117.68 (2.85)
** Gigantocellular ret. n.**	87.09 (0.71)	88.32 (1.23)
**Cerebellum**		
** Granule cell layer**	128.45 (1.98)	126.64 (1.91)
** Molecular cell layer**	130.08 (1.90)	127.05 (1.62)
** Cerebellar n.**		
**Interposed**	119.34 (1.39)	117.96 (1.38)
**Lateral (Dentate)**	131.43 (1.36)	130.17 (1.52)
**Medial (Fastigial)**	90.65 (1.71)	91.17 (1.78)
** Vestibular n.**		
**Lateral (Deiters)**	89.73 (1.06)	90.16 (2.33)
**Medial vestibular n.**		
**-Magnocellular part**	112.01 (0.91)	111.83 (3.16)
**-Parvocellular part**	138.46 (1.52)	140.51 (1.98)

Percentage of optical densities of cytochrome oxidase activity staining after everolimus treatment in the telencephalon, the diencephalon, the mesencephalon, the metencephalon, the myelecephalon and the cerebellum.

Data are means +SEM. (Student *t* test, *p<.05). (a, area; ant, anterior; Diag, diagonal; horiz, horizontal; genic, geniculate; mesenc, mesencephalic; n, nucleus; ret, reticular; vert, vertical).

### No emotional and cognitive alteration after everolimus treatment

#### Emotional behavior

Seven days after the end of the treatment period, the percentage of open-arm entries in the elevated plus maze (See Material and Methods S1) was not significantly different between the 2 treatment groups, suggesting that everolimus did not affect anxiety-like behavior (t_24_ = 1.66, *p*>.05; Fig. S1A). In the forced swimming test, the immobility duration was not statistically different between groups, indicating that everolimus did not modify depressive-like behavior (t_24_ = 0.65, *p*>.05; Fig. S1B).

#### Spatial learning and memory

**Figure 3:**
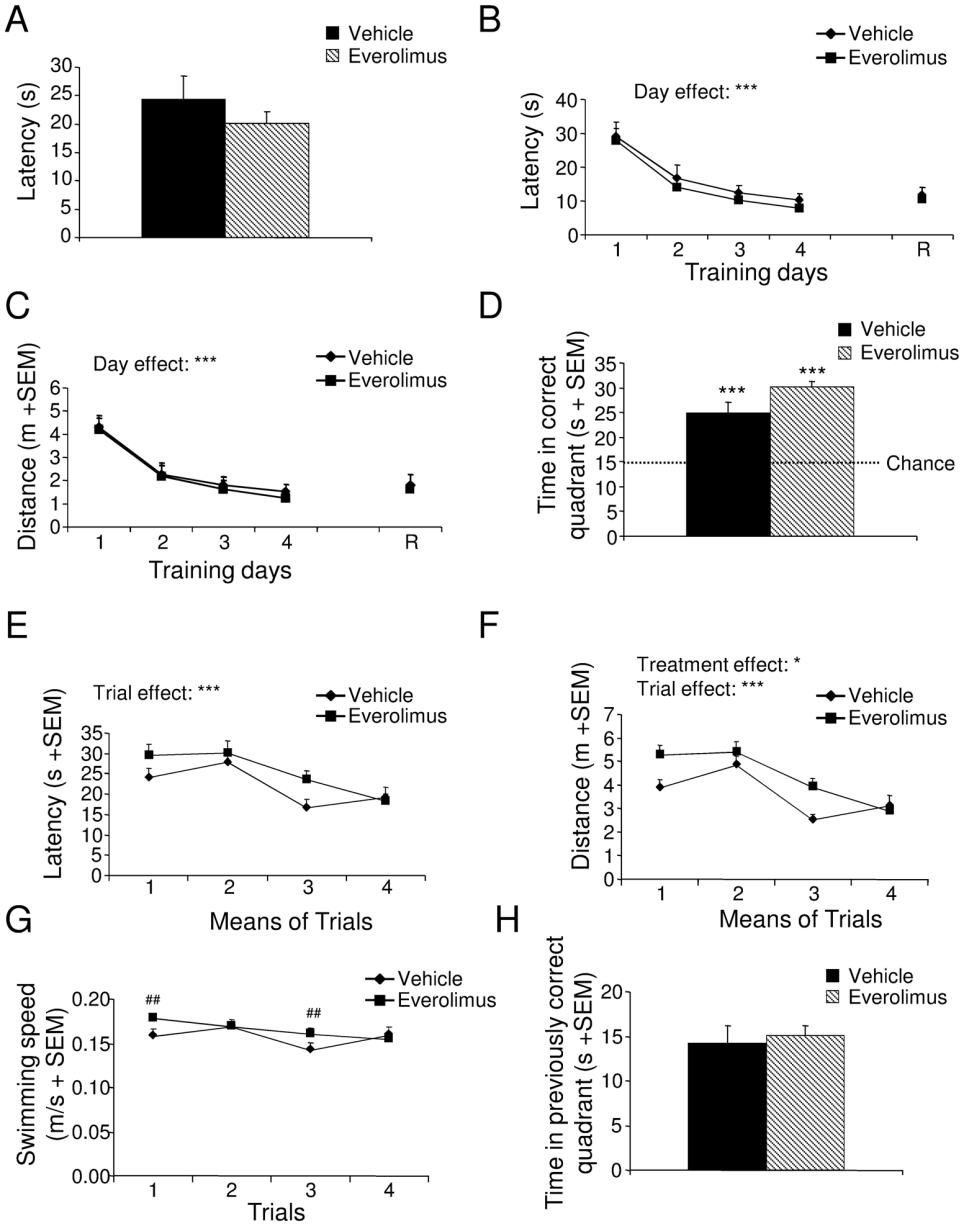
Spatial learning and memory, and behavioral flexibility of mice treated with vehicle or everolimus in the Morris water maze test. (A) Motivation and visuo-motor abilities after vehicle or everolimus treatment (Student *t* test, *p*>.05). (B and C) During the training and retrieval (R) phases, latency (B) and distance crossed (C) after vehicle or everolimus treatment (ANOVA, Day effect ***p<.001). (D) During the probe test, time spent by animals of both groups in the quadrant where the platform was located during the training phase (χ^2^, ***p<.001 *vs* chance). During the transfer phase, escape latency (E), distance crossed (F), and swimming speed (G) in vehicle- or everolimus-treated mice (ANOVA, Treatment effect *p<.05, Trial effect *** *p*<.001 followed by LSD post hoc ^##^ *p*<.01). (H) During the 1st day of the transfer phase, time spent in the previously correct northwest quadrant after vehicle or everolimus treatment (Student *t* test, *p*>.05). Data are means +SEM.

During the familiarization phase of the Morris water maze test, swimming speed (t_24_ = −0.69, *p*>.05; not shown), distance crossed (t_24_ = 0.49, *p*>.05; not shown) and time taken to find the emerged platform (t_24_ = 0.93, *p*>.05; Fig. 3A) were not significantly modified by treatment, indicating that motivation and visuo-motor ability were unaffected by everolimus. During the acquisition phase, everolimus did not alter spatial learning performance 15 days after the end of the treatment period, as the escape latency (F_1,24_ = 0.94, *p*>.05; Fig. 3B) and distance crossed (F_1,24_ = 0.32, *p*>.05; Fig. 3C) were not significantly different between groups. Performances improved significantly as the test was repeated (escape latency F_3,72_ = 18.38, *p*<.001 and distance crossed F_3,72_ = 19.51, *p*<.001). During the probe test, all mice spent significantly more time in the previously correct quadrant than predicted by chance (vehicle: X^2^_11_ = 117.67, *p*>.001; everolimus: X^2^_13_ = 231.80, *p*<.001; Fig. 3D). During the retrieval phase, everolimus-treated mice did not display spatial memory performance impairment, as their escape latency (t_24_ = 0.49, *p*>.05; Fig. 3B) and distance crossed (t_24_ = 0.40, *p*>.05; Fig. 3C) did not differ significantly from those of vehicle mice.

During the transfer phase of the Morris water maze test, animals improved their performances across trials (escape latency: F_3,72_ = 9.76, *p*<.001; distance crossed F_3,72_ = 14.99, *p*<.001; Figs. 3E and 3F). Learning plasticity, assessed by daily modification of the platform location, was unaffected by treatment; escape latency before finding the hidden platform was similar between groups (F_1,24_ = 3.36, *p*>.05; Fig. 3E). However, the distance crossed by everolimus-treated mice was significantly increased compared with vehicle mice (F_1,24_ = 6.08, *p*<.05; Fig. 3F). ANOVA assessment revealed a significant treatment × trial interaction in terms of swimming speed (F_3,72_ = 3.69, *p*<.05). LSD post hoc analyses indicated that everolimus-treated mice swam faster than vehicle mice during the first and third trials (*p*<.01; Fig. 3G). During day 1 of the transfer phase, everolimus did not alter the time spent in the quadrant that was correct during the training phase (t_24_ = −0.39, *p*>.05; Fig. 3H), suggesting the absence of behavioral perseveration.

#### Spontaneous activity and recognition memory

Everolimus did not modify spontaneous locomotor and vertical activities. The distance crossed and the leaning number were not significantly different between treatment groups (t_24_ = 0.63, *p*>.05 and t_24_ = 0.39, *p*>.05, respectively; Figs. S1C and S1D). In the object recognition test, animals of both groups detected novelty, with longer exploration durations of the novel object *vs* the familiar ones (vehicle: t_11_ = −2.90, *p*<.05; everolimus: t_13_ = −4.51, *p*<.001; Fig. S1E). No significant difference in exploration durations between groups was detected for either novel (t_24_ = −0.37, *p*>.05) or familiar objects (t_24_ = −0.23, *p*>.05; Fig. S1E). For details of procedures, please refer to Material and Methods S1.

### Hippocampal neural precursor cell proliferation *in vivo* and *in vitro*

**Figure 4:**
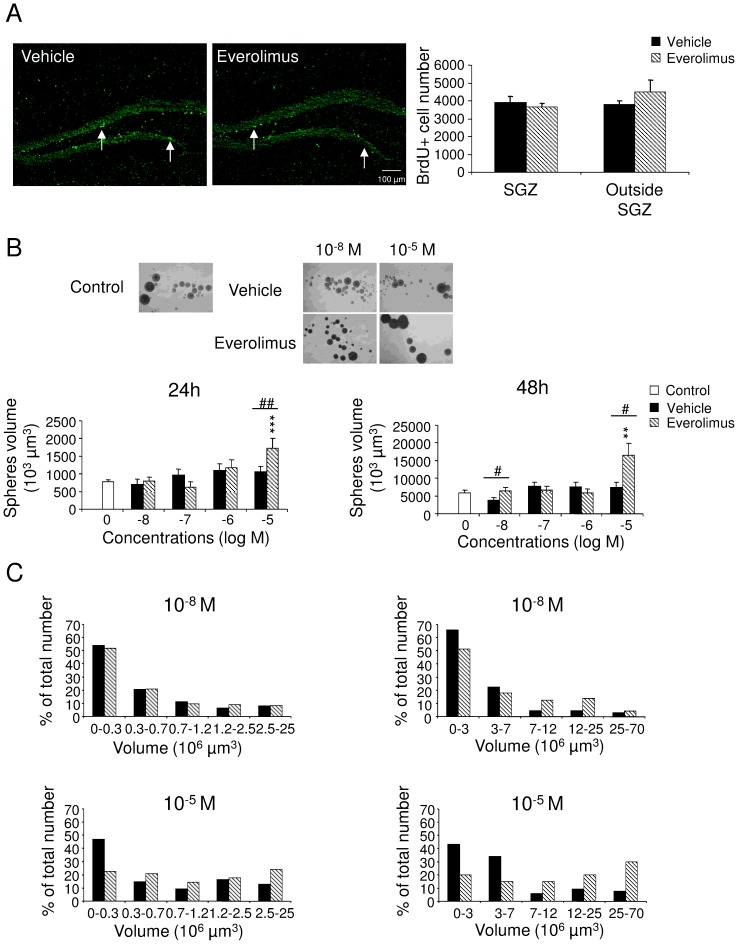
Effect of everolimus on precursor cell proliferation *in vivo* and *in vitro.* (A) Effect of *in vivo* administration of everolimus on neural precursor cell proliferation in the hippocampus. Left, BrdU-positive cells (intense green cells, white arrows) were counted in the subgranular zone (SGZ) and the remaining part of the dentate gyrus (outside SGZ). Bregma −2.16 mm; Scale bar, 100 µm. Right, Number of BrdU^+^ cells in the SGZ or outside SGZ (Mann whitney U tests, *p*>.05). (B and C) Effect of increasing concentrations of vehicle and everolimus on neural stem cell growth in culture. (B) *Upper*, Phase-contrast image of floating neurospheres at 24 hours after treatment with vehicle and everolimus (10^−8^ and 10^−5^ M). *Below*, Neurosphere volume in the absence (control: 0) and presence of vehicle and everolimus after 24 hours and 48 hours of treatment (Kruskal-Wallis ANOVA followed by Dunn's tests ***p<.001 vs 0; Mann Whitney U-tests, ^#^ *p<.05,* ^##^ *p<.01* everolimus *vs* vehicle. Data represent means +SEM. (C) Proportion of neurospheres according to different categories of volumes. The increase of neurosphere mean volume might be explained by an increased proportion of bigger neurospheres and a decreased proportion of smaller neurospheres associated with everolimus treatment.

The learning and memory tasks used are dependent on hippocampal functions, thus we first assessed whether everolimus would have affected neural precursor cell proliferation and survival. As shown in Fig. 4A, everolimus treatment over a 14-day period did not modify the number of BrdU-positive cells, either in the SGZ (U = 6, *p*>.05) or outside this area (U = 6.5, *p*>.05). To verify whether targeted therapies altering the mTOR pathway may affect neural stem cells (NSC), everolimus was directly tested on NSC in culture. NSC cultured from mouse newborn neuroepithelium formed (after 3 passages) small aggregates likely containing less than 10 cells (Fig. 4B). Neurospheres became bigger at day 2 (24 hours) and day 3 (48 hours) after the third passage. Everolimus (10^−5^ M) led to a significant increase of mean neurosphere volume compared with vehicle (10^−5^ M, U = 2435.5, *p*<.01) and compared with control conditions at 24 hours (H_5_ = 32.95, *p*<.001, followed by Dunn's test *p*<.001) and at 48 hours (U = 333.5, *p*<.05; H_5_ = 10.72, *p*<.05, followed by Dunn's test *p*<.01, respectively) (Fig. 4B). However, the increase in mean volume of neurospheres induced by everolimus at higher doses might be explained by a reduced proportion of neurospheres with smaller volumes and an increased proportion of neurospheres with larger volumes (Fig. 4C). In agreement, everolimus did not modify cell cycle progression of NSC as shown in Fig. S2.

### Vascular component density

**Figure 5:**
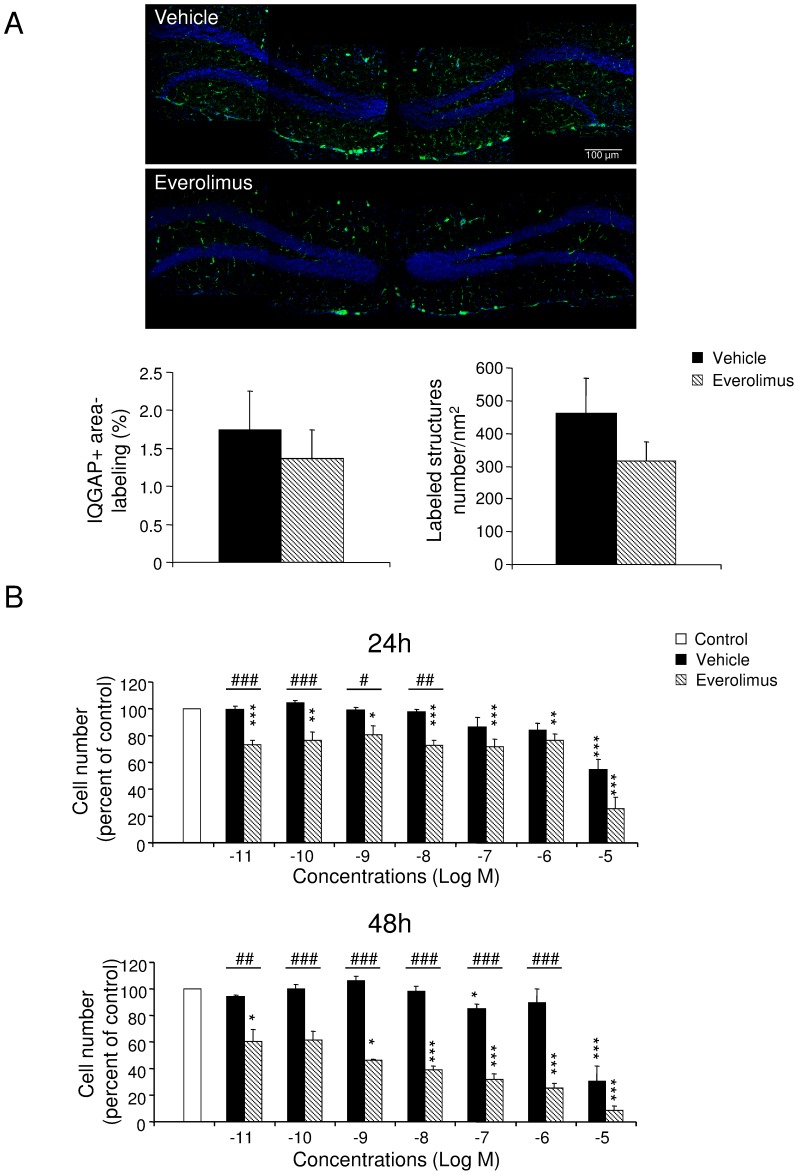
Vascular component density in hippocampus and endothelial cell proliferation *in vitro.* (A) Vascular density in the dentate gyrus of the hippocampus. *Upper*, IQGAP1-immunolabeled vascular niches in the dentate gyrus were illustrated from brain slices of vehicle- and everolimus-treated mice (Bregma −1.68 mm). Scale bar, 100 µm. *Below*, Mean IQGAP1-positive surface labeling and number of branches/nm^2^ (+SEM) in the dentate gyrus (Mann-Whitney U-tests, *p*>.05). (B) Effects of increasing concentrations of vehicle and everolimus on the bEND.3 endothelial cell growth, as evaluated by endothelial cell number after 24 hours and 48 hours of treatment with vehicle or everolimus. Values are normalized to the mean number of cells in the control condition (white bar). Effect of everolimus, Kruskal-Wallis ANOVA followed by Dunn's tests *p<.05, **p<.01, ***p<.001 *vs* control; Effect of everolimus compared with vehicle (Mann-Whitney tests, #*p*<.05, ##*p*<.01, ###*p*<.001).

To visualize brain vessel architecture, we studied the labeling of IQGAP1 scaffolding protein, known to be expressed in adult mouse brain by parenchymal endothelium, ventricular epithelial ependymal cells, and neural progenitor and neuronal precursor cells in the SGZ [42]. Prolonged everolimus treatment did not significantly diminish IQGAP1-positive structure area (U = 4, p>.05) and number of labeled structures (U = 1, p>.05; Fig. 5A). We then investigated the direct effect of everolimus on endothelial cells by using the bEND.3 cell line [43], [44] (Fig. 5B). We showed that everolimus from 10^−9^ to 10^−5^ M inhibited cell survival compared with the control condition at 24 hours (H_7_ = 63.21, p<.001, followed by Dunn's tests p<.05) and at 48 hours (H_7_ = 70.12, p<.001, followed by Dunn's tests p<.05). Compared with vehicle, everolimus decreased cell numbers at concentrations comprising between 10^−11^ M and 10^−8^ M at 24 hours (U>0, p<.05) and at concentrations comprising between 10^−11^ M and 10^−6^ M at 48 hours (Fig. 5B) (U>0, p<.01). At 10^−5^ M, vehicle led to reduced cell numbers compared with control at 24 hours (H_7_ = 28.90, p<.001, followed by Dunn's test p<.001) and 48 hours (H_7_ = 32.06, p<.001, followed by Dunn's test p<.001).

## Discussion

As cancer treatments are constantly improving, *via* the more widely used targeted therapies, more efforts have to be made to implement prevention and management of side effects in delivering cancer therapy. In general, molecular-targeted therapies can be considered relatively safe, compared with chemotherapy. However, inhibitors of the p-Akt/mTOR pathway such as everolimus, although well tolerated, are reported to be associated with fatigue, hyperglycemia, hypertriglyceridemia, hypercholesterolemia, gastrointestinal syndromes, anorexia, rash, mucositis or headache [25], [26]. More specifically, CNS side effects have been also described, including headache/migraine (up to 30%), convulsions (up to 29%), dysgeusia (up to 19%), dizziness (up to 14%) or paresthesia (up to 5%) (26). In addition, the inhibition of the mTOR pathway was described affecting cognitive functions [18], [45], [46]. Thus, fatigue in addition to other observed and/or on studied CNS symptoms should greatly affect quality of life and wellbeing, and can adversely affect physical and mental functioning such as cognitive functions. Understanding the potential impact or the safety of systemic administration of everolimus on cognitive function and brain neural cell activity should provide key insights to better define solutions for planning, guiding, and monitoring this cancer therapy.

As such, we took advantage of a validated animal model as an original sensitive neuropsychological paradigm [28], which is an unique way to explore direct causal links between targeted therapy and disorders in learning and memory processes, behavioral flexibility and neural cell activity. Previous experiments indicated that antibodies recognizing mTOR components present an ubiquitous distribution in the CNS and there is scattered expression of phosphorylated mTOR in the cortex, hippocampus and hypothalamus [7].

In studies of patients with solid tumors treated with chemotherapy, moderate cognitive dysfunction has been reported in 15%–50% of cases [1]. Such impairment mainly affects executive functions, resulting in reduced concentration levels and slowness of response and disorganization, but it may also include memory deficits, affecting recently learned information and spatial memory [1]. Previous studies have shown that consolidation relies on molecular mechanisms involving mTOR signaling pathway [47] and that infusion of mTOR inhibitor into the hippocampus during a short period around training impairs long-term memory in the object recognition task or the Morris water maze test [18], [45], [46]. In the current study, the potential lasting effect of a chronic oral administration of everolimus was evaluated by testing mice several days after the treatment was ended. Everolimus induced no changes in emotional reactivity and did neither alter spatial learning/memory performances/learning flexibility nor memory in the object recognition task. These data differ from previous studies on chemotherapy-treated animals, which demonstrated deficits in behavioral flexibility and object recognition memory or longer object exploration [28], [48]–[51]. We demonstrated that systemic administration of everolimus did not modify spatial learning and memory or executive functions that would persist at one to four weeks after the end of the treatment completion. This observation also appears divergent from data showing that mTOR inhibitors such as rapamycin alter cognitive functions [18], [45], [46]. This discrepancy may be explained by the direct infusion of rapamycin into the hippocampus parenchyma in the study from Dash *et al.* and Myskiw *et al.* [18], [45] compared with the evaluation of the learning and memory performances after systemic administration of everolimus, only after and not during the treatment in the present study. Also, it can be mentioned that everolimus was suggested to slowly penetrate brain as evidenced by inhibition of S6 kinase phosphorylation in the cortex and the striatum only when 20 µmol/kg dose was used [52], [53]. These data may suggest that the dose of everolimus (5 mg/kg) used here remained rather low to cross the blood brain barrier and accumulate into various brain area.

Our study also verified the potential impact of the mTOR pathway inhibitor on hippocampal precursor neural cell proliferation during acute treatment. Everolimus did not affect cellular proliferation in the dentate gyrus of the hippocampus when evaluated immediately after the last day of treatment. This suggests that mTOR inhibition may not be associated with alteration of NSC mitogenesis within the subgranular zone in particular and/or that two weeks of everolimus should not control NSC within vascular niches in the hippocampal neurogenic area. Interestingly, we confirmed *in vitro* that everolimus from 10^−8^ to 10^−6^ M did not affect NSC cell cycle and neurosphere growth. Accordingly, previous data suggest that mTOR inhibition may, indeed, have no impact on self-renewal [54] but would likely prevent the growth of neural stem/progenitor cells more particularly under hypoxic [55] or kainate-induced seizure [56] conditions. Thus, under normoxic experimental conditions, the mTOR pathway would not be sufficiently activated and involved in NSC cell cycle, supporting the absence of observed effect of everolimus on NSC proliferation.

Targeted therapies based on small molecule inhibitors directed against the mTOR pathway would alternatively impede synaptic plasticity and/or cerebrovascular function. We thus hypothesized that everolimus would have modified the vascular architecture and the organization of vascular niches. The immunolabeling of IQGAP-1 in the hippocampus revealed no significant alteration of the vascular densities or branching in brain slices from everolimus-treated mice, suggesting that everolimus may not interfere with vascular architecture. To clarify this aspect, we tested whether everolimus may interact *in vitro* with proliferation/survival of brain endothelial rodent bEND.3 cells and showed altered cell survival compared with control conditions. These findings were consistent with the results of Barilli *et al.*, [57] who demonstrated that prolonged exposure to rapamycin affects *in vitro* human umbilical vein endothelial cell and human aortic endothelial cell viability at concentrations equal to or larger than 10 nM, increasing both necrotic and apoptotic parameters. This drastic diminution of endothelial cell number indicates that everolimus can directly affect the endothelial function at least *in vitro*. Together, the absence of *in vivo* effects one day after the end of the treatment, on neural cell division and vascular organization in the hippocampus would suggest that everolimus does induce alterations of some key brain areas (such as the as hippocampus) involved in cognitive functions.

We can still emphasize that everolimus led to a delayed and persistent reduction in weight gain from the last day of the treatment period. A weight loss has been reported in patients [25], [58] and in animal models [22], [33], [59] treated with everolimus. For instance, in the study of Farb [59], everolimus-treated animals, experience weight loss (10%) and loss of appetite but appeared healthy otherwise, according to the authors. Moreover, Mabuchi et al. [22] reported that in transgenic mice developing bilateral ovarian serous adenocarcinoma, 5 mg/kg/twice a week led to a 10% lower weight than vehicle mice beginning from the third week of treatment. After 5 weeks of treatment, they mentioned histopathological analyses of liver, spleen, pancreas, kidney, small and large intestine, uterus and ovaries, and conclude with no evident toxic changes [22]. As mTOR signaling pathway plays an important role in homeostasis in visceral organs and brain [2], [60], weight loss described after everolimus treatment could be associated with a skeletal muscle atrophy caused by a protein synthesis alteration in muscles [58] and/or inhibition of fat accumulation [61], but also with troubles with feeding behavior. Accordingly, rats treated with a single dose of peripheral rapamycin maintained a lower body weight whereas it did not induce malaise of illness and this effect seems to be mediated at least partially by the brain [62]. Altogether, mTOR inhibition by everolimus would mimick deficiency in nutrients and growth factors and may alter whole body energy homeostasis and food intake in particular through gastric, pancreatic, adipocytes and/or cerebral functions [63]–[67].

**Figure 6:**
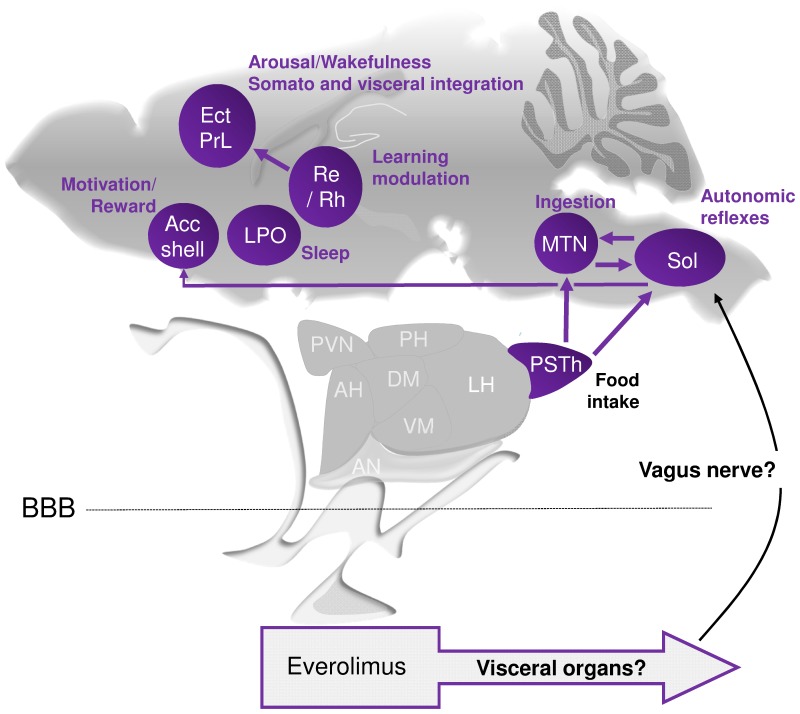
Schematic representation of brain areas presenting cytochrome oxidase activity modifications in mice treated with everolimus. Everolimus does not induce alteration of the phosphorylated P70S6K in the studied brain areas. It is here hypothesized that everolimus direct action on visceral organs would impact some cerebral areas through the vagus nerve. Among the brain areas with CO activity modifications are the solitary nucleus (Sol) as the principal recipient of visceral information conveyed by the vagus afferents and connected with the autonomic nervous system, motor nuclei of cranial nerves, nuclei in the brainstem and hypothalamus; the parasubthalamic nucleus (PSTh) within the hypothalamus, and motor trigeminal nucleus (MTN) involved in ingestion; the accumbens (Acc) shell important for reward and motivation processes; the preoptic area (LPO) regulating sleep/wake state; and the thalamic reuniens (Re) and rhomboid (Rh) nuclei and cortical areas that integrate arousal, somatosensory and visceral information, and participate to the modulation of learning and memory, and maintenance of arousal and wakefulness. Thus brain areas with modified CO activity are involved in the integration of autonomic and neuroendocrine information important for regulation of food intake, weight, metabolic, motivational processes, and arousal, that could be associated with symptoms suggestive of fatigue and energy homeostasis alterations. Arrows in purple show connections between brain areas metabolically modified. AH, anterior hypothalamic area; BBB, brain blood barrier; DM, dorsomedial hypothalamic nucleus; Ect, ectorhinal cortex; LH, lateral hypothalamic area; PH, posterior hypothalamic nucleus; PrL, prelimbic cortex; PVN, paraventricular hypothalamic nucleus; VM, ventromedial hypothalamic nucleus.

In order to investigate the global impact of everolimus on cerebral functions, a mapping of regional brain cell activity (cytochrome oxidase staining) was performed immediately following the two-week long period treatment. As summarized in Figure 6, selective alterations of cytochrome oxidase activity were detected in regions involved in energy homeostasis and weight regulation (parasubthalamic area of the hypothalamus), food intake and ingestion (hypothalamus, motor trigeminal nucleus, solitary nucleus), reward and motivation (accumbens shell), learning and memory modulation (thalamus, cerebral cortex), and sleep/wakefulness control and arousal (preoptic area, cerebral cortex) [68]–[72]. However, the level of activation of the P70S6K (phospho-P70S6K) within these cerebral areas were not modified after everolimus treatment, suggesting a rather direct peripheral mTOR inhibition leading, in turn, to indirect brain alterations. For instance, the hepatic vagus nerve senses glucose production in the liver and the vagal afferents can be activated by the gastrointestinal system, then sending signals to the CNS and more particularly the solitary nucleus. We may propose that under inhibition of mTOR by everolimus, the use of glucose by the muscle, fat, the liver or the gut produces glucose and ketones leading to activation of the nucleus of the solitary tract through the vagus nerve (Fig. 6). The parasubthalamic area, through its connections with the solitary nucleus, the accumbens nucleus, or the motor trigeminal nucleus, is positioned to influence feeding behavior and weight [73]–[76]. In particular, the parasubthalamic nucleus is suggested to be involved in the restraining of food intake in some situations such as conditioned taste aversion and indispensable amino acids deficiency [76], [77]. Thus, autonomic functions, orofacial control, feeding and motivation may be particularly affected during cancer treatment with everolimus, but we cannot conclude whether these symptoms would potentially last more than 1 week after the end of the treatment completion.

In conclusion, chronic systemic treatment with the mTOR inhibitor everolimus did not affect emotional reactivity, spontaneous activity, spatial learning and memory, behavioral flexibility, and object recognition memory when evaluated 7 to 29 days after the end of the treatment. Also, this therapy provided *via* oral administration did not modify neural precursor cell proliferation or vascular component distribution in the hippocampus. However, cerebral metabolism, in selective regions connected with the solitary nucleus and the hypothalamus and related to weight regulation and motivation, was altered by acute administration of everolimus. This evaluation of cognitive functioning and brain metabolic activity following everolimus treatment might help to elucidate the physiopathology of the neurological alterations observed among cancer patients treated with targeted therapies such as mTOR inhibitors.

## Supporting Information

Figure S1 **Emotional reactivity, spontaneous activity, and object recognition memory of mice treated with everolimus or vehicle.** The anxiety-like behaviors evaluated in the elevated plus maze (A), the depressive-like behaviors evaluated in the forced swim test (B), and the spontaneous locomotor (C) and vertical activity (D) were not modified by treatment (Student *t* test, *p*>.05). (*E*) Everolimus did not modify object recognition memory performances, and mice in both groups detected novelty (Student *t* test, *p<.05, **p<.01, *vs* familiar). Data are means +SEM.
(PDF)

Figure S2 **Cell cycle evaluation of neural stem cells after everolimus treatment.** Effects of increasing concentrations of vehicle or everolimus on neural stem cell cycle after 24 hours of treatment. Cell cycle analysis following propidium iodide intercalation into the cellular chromatin was performed by flow cytometry in the absence or presence of vehicle or everolimus (10^−8^–10^−5^ M). Data are represented as relative fluorescence intensity of sub G1, G1, S and G2/M-phase population in a 2-dimensional cytometry profile. At 10^−5^ M, the number of cells in the sub-G1 phase increased in both vehicle and everolimus conditions.
(PDF)

Figure S3 **Chronology of behavioral and** ***ex vivo*** **studies following the treatment period.** (A) From day 0 to day 13, mice were treated with vehicle or everolimus, with a 7-day interval before initiation of behavioral evaluations on day 20, which continued for 23 days. (B) 24 hours subsequent to the 14 days treatment period, mice brains were removed and processed to reveal cytochrome oxidase activity. (C) After 6 injections of 5-bromo-2-deoxyuridine (BrdU), and 24 hours following the last treatment with vehicle or everolimus, mice brains were removed and immunohistochemically labeled for neural cell proliferation and vascular density.
(PDF)

Material and Methods S1 (DOC)
