# Supplementary figures and images for: Evaluation of the Impact of the Cancer Therapy Everolimus on the Central Nervous System in Mice
Source: PLoS One. 2014 Dec 1;9(12):e113533. doi: 10.1371/journal.pone.0113533 (PMC4250083; doi:10.1371/journal.pone.0113533)

A

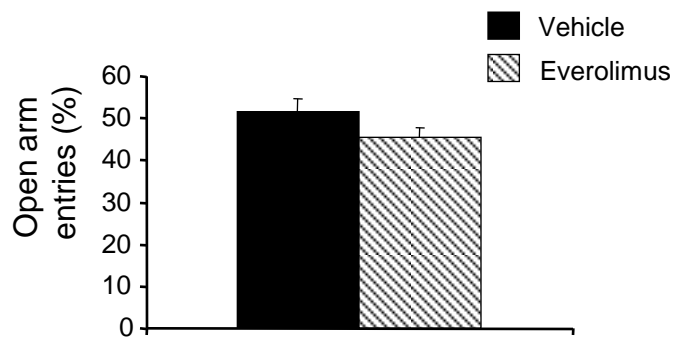

B

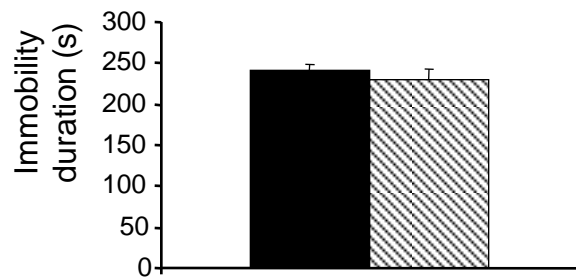

C

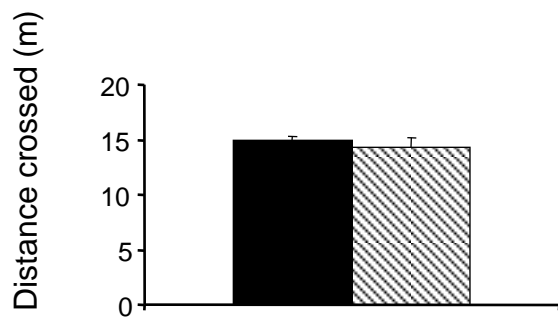

D

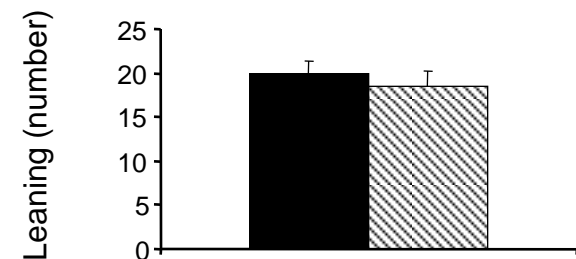

E

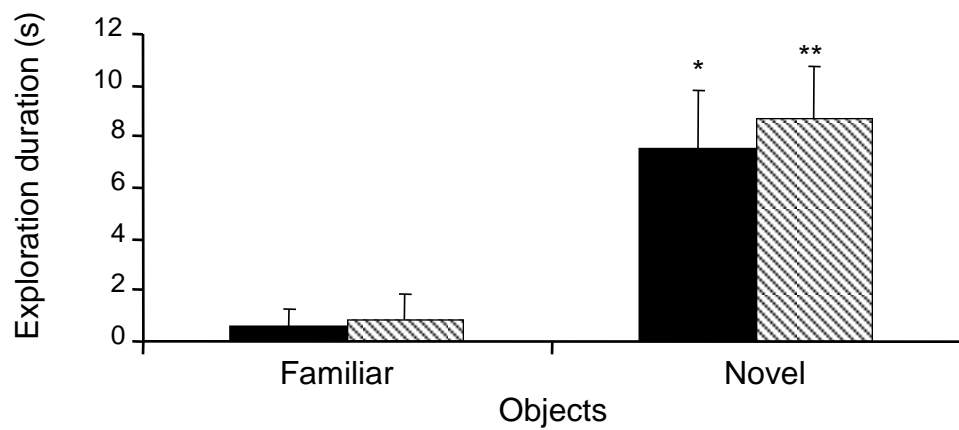

Supplement: Figure S1 — Emotional reactivity, spontaneous activity, and object recognition memory of mice treated with everolimus or vehicle. The anxiety-like behaviors evaluated in the elevated plus maze (A), the depressive-like behaviors evaluated in the forced swim test (B), and the spontaneous locomotor (C) and vertical activity (D) were not modified by treatment (Student t test, p>.05). (E) Everolimus did not modify object recognition memory performances, and mice in both groups detected novelty (Student t test, *p<.05, **p<.01, vs familiar). Data are means +SEM. (PDF) [file pone.0113533.s001.pdf]

Control

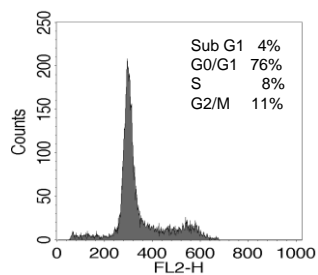

Vehicle

 $10^{-8}$  M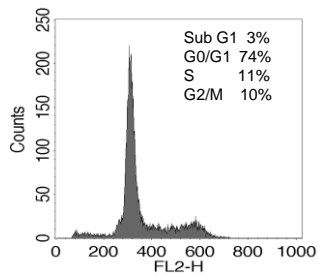

Everolimus

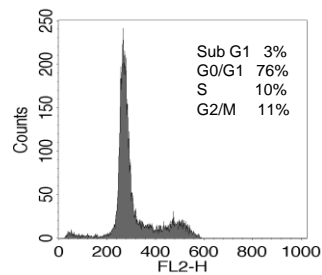 $10^{-7}$  M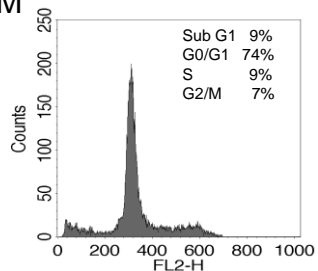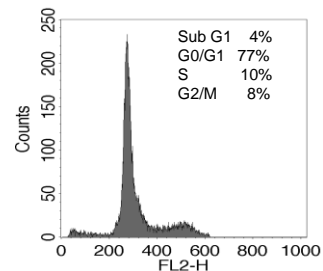 $10^{-6}$  M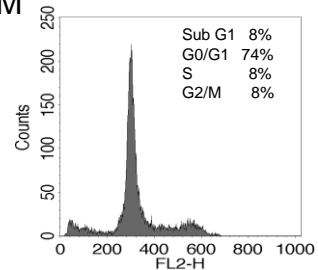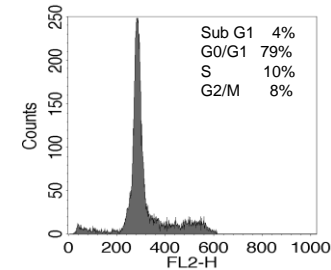 $10^{-5}$  M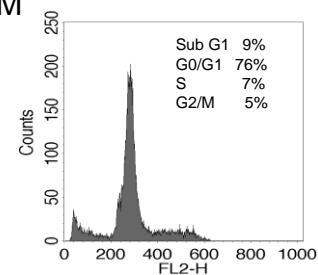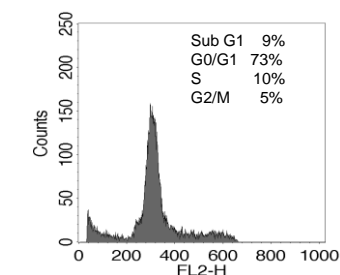

Supplement: Figure S2 — Cell cycle evaluation of neural stem cells after everolimus treatment. Effects of increasing concentrations of vehicle or everolimus on neural stem cell cycle after 24 hours of treatment. Cell cycle analysis following propidium iodide intercalation into the cellular chromatin was performed by flow cytometry in the absence or presence of vehicle or everolimus (10−8–10−5 M). Data are represented as relative fluorescence intensity of sub G1, G1, S and G2/M-phase population in a 2-dimensional cytometry profile. At 10−5 M, the number of cells in the sub-G1 phase increased in both vehicle and everolimus conditions. (PDF) [file pone.0113533.s002.pdf]

A

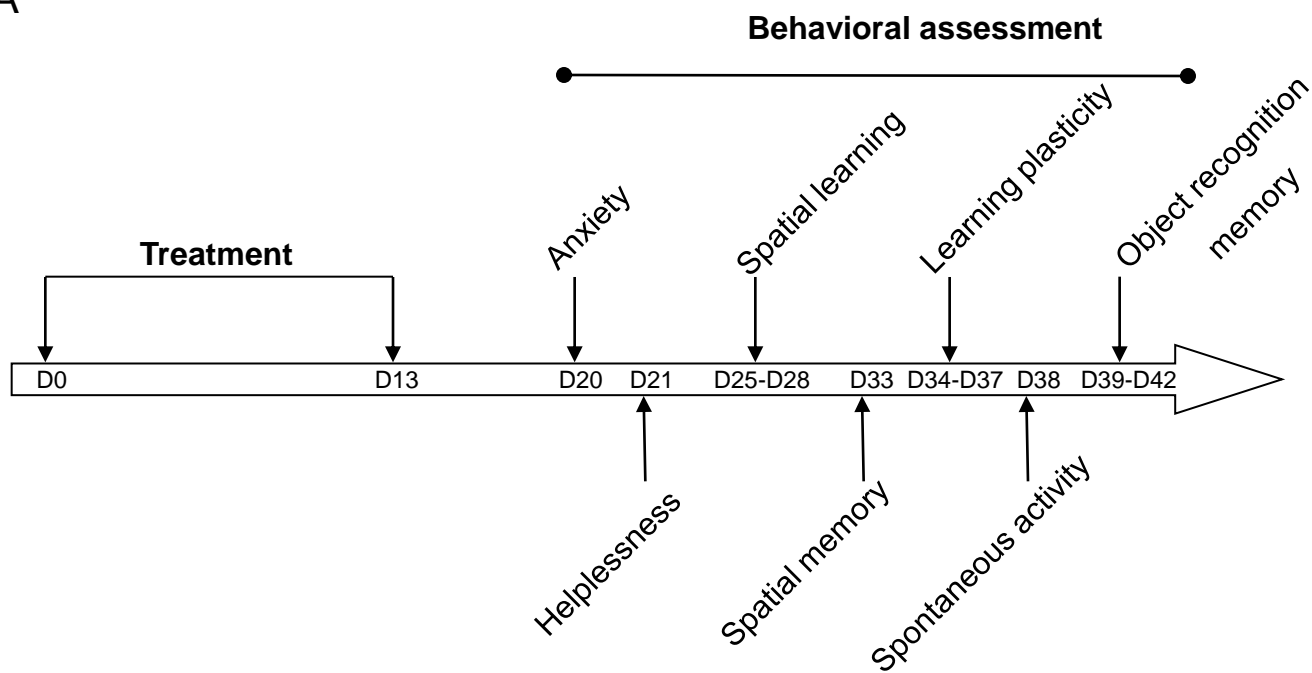

B

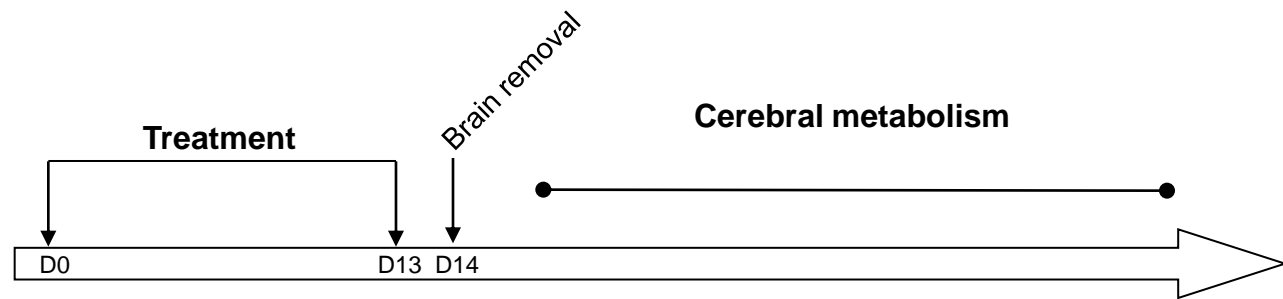

C

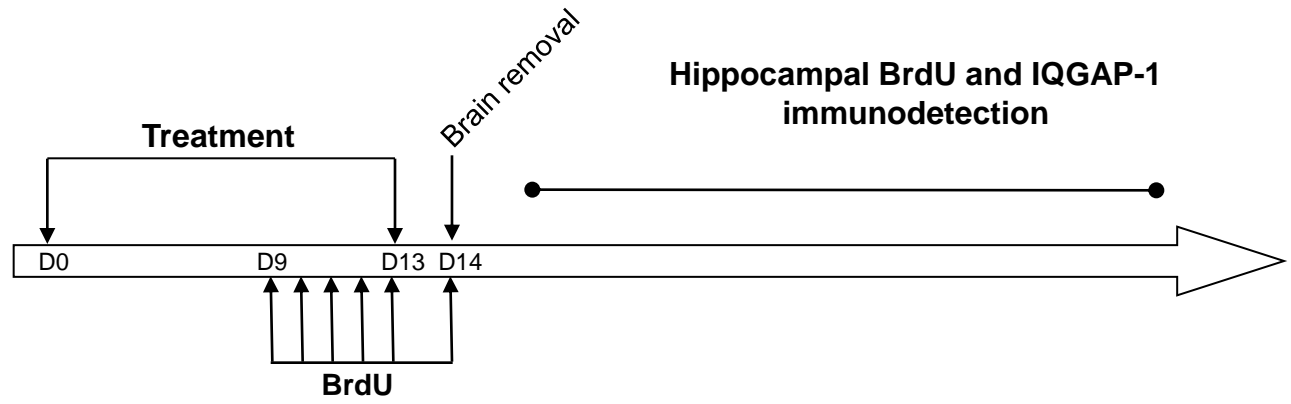

Figure S3

Supplement: Figure S3 — Chronology of behavioral and ex vivo studies following the treatment period. (A) From day 0 to day 13, mice were treated with vehicle or everolimus, with a 7-day interval before initiation of behavioral evaluations on day 20, which continued for 23 days. (B) 24 hours subsequent to the 14 days treatment period, mice brains were removed and processed to reveal cytochrome oxidase activity. (C) After 6 injections of 5-bromo-2-deoxyuridine (BrdU), and 24 hours following the last treatment with vehicle or everolimus, mice brains were removed and immunohistochemically labeled for neural cell proliferation and vascular density. (PDF) [file pone.0113533.s003.pdf]
