# Supplementary material for: Evaluation of the Impact of the Cancer Therapy Everolimus on the Central Nervous System in Mice
Source: PLoS One. 2014 Dec 1;9(12):e113533. doi: 10.1371/journal.pone.0113533 (PMC4250083; doi:10.1371/journal.pone.0113533)
Supplement: Material and Methods S1 — (DOC) [file pone.0113533.s004.doc]

**Supporting Material and Methods**

**Emotional reactivity**

Anxiety-related behaviors were evaluated using the elevated plus maze. This test allows determination of emotional reactivity in rodents by means of a conflict between secure (enclosed arms) and aversive (open arms) parts of the maze (1). Arms measured 25 cm long and 5 cm wide, with the 2 opposing closed arms enclosed by 19.5 cm height walls and the 2 opposing open arms bordered with 0.5 cm edges. The apparatus, made of ivory perspex (Intellibio®, Seichamps, France), was elevated 41.5 cm above the floor and placed in a dimly lit room, each open arm being illuminated at 150 lux. Each mouse was placed in the center of the maze with its head facing an enclosed arm and video tracked with Anymaze (Stoelting®, Dublin, Ireland). The number of open and closed arm entries (4 paws criterion) were noted during 5 minutes, and the percentage of entries into open arms calculated.

Depressive-like behavior was evaluated with the forced swim test (2), a standard procedure used to evaluate the antidepressant activity of pharmacological compounds. Mice were placed into a cylinder (diameter 17.2 cm) filled to a height of 15 cm with 25°C tap water for 6 minutes (3). The mobility time (excluding movement necessary to keep the head above water or to float) was noted, and the immobility duration, indicative of the behavioral despair of mice, was calculated.

**Spontaneous activity and recognition memory**

Spontaneous locomotor and vertical activities were assessed in an open-field wooden box 30 × 30 × 30 cm, 30 lux in the center. Animals were placed in the apparatus for 5 minutes and the distance crossed measured. This box was also used to evaluate object recognition memory (4, 5). During 3 consecutive days (sample session), 3 identical objects (plastic bottle cap) were placed on the floor of the apparatus and mouse exploration behavior (sniffing, leaning and climbing) was evaluated for 5 minutes. On the fourth day (test session), 1 familiar object was replaced with a novel object, and exploration durations of both familiar and novel objects were measured for 5 minutes. To take into account the exploration behavior of each group, the difference between the novel object exploration duration during the 5-minute test session and that of the last day of the sample session was calculated.

**Cell cycle analysis**

Neural stem cells (NSC) were plated in 25 cm^2^ flasks (Techno plastic products AG, Trasadingen, Switzerland). When NSC reached >200 µm in diameter, they were incubated in the absence or presence of vehicle or everolimus (10^-8^-10^-5^ M) for 24 hours. Cell cycle analysis was done by DNA staining with propidium iodide by flow cytometry. Briefly, after treatment, cells were trypsinized, harvested in phosphate-buffered serum, and fixed in ice cold 70% ethanol for 2 hours. Staining was done with 0.6 mg/mL RNase (30 minutes, RT; Sigma-Aldrich, Saint-Quentin Fallavier, France) and 50 µg/mL propidium iodide (30 minutes, RT; Sigma-Aldrich, Saint-Quentin Fallavier, France). Cell cycle status was quantified by FacsCalibur flow cytometer (BD Biosciences, Le pont de Claix, France) using an excitation laser set at 480 nm and a detection wavelength of 670 nm (FL3), to avoid detection of FI excitation.

**References**

1. Lister, R. G. (1987) The use of a plus-maze to measure anxiety in the mouse. Psychopharmacology (Berl) 92: 180-185

2. Porsolt, R. D., Bertin, A., and Jalfre, M. (1977) Behavioral despair in mice: a primary screening test for antidepressants. Arch Int Pharmacodyn Ther 229: 327-336

3. Bachli, H., Steiner, M. A., Habersetzer, U., and Wotjak, C. T. (2008) Increased water temperature renders single-housed C57BL/6J mice susceptible to antidepressant treatment in the forced swim test. Behav Brain Res 187: 67-71

4. Dere, E., Huston, J. P., and De Souza Silva, M. A. (2005) Episodic-like memory in mice: simultaneous assessment of object, place and temporal order memory. Brain Res Brain Res Protoc 16: 10-19

5. Roy, V., and Chapillon, P. (2004) Further evidences that risk assessment and object exploration behaviours are useful to evaluate emotional reactivity in rodents. Behav Brain Res 154: 439-448
